# Hypervirulent *Klebsiella pneumoniae* of Lineage ST66-K2 Caused Tonsillopharyngitis in a German Patient

**DOI:** 10.3390/microorganisms9010133

**Published:** 2021-01-08

**Authors:** Kathleen Klaper, Sebastian Wendt, Christoph Lübbert, Norman Lippmann, Yvonne Pfeifer, Guido Werner

**Affiliations:** 1 Division Nosocomial Pathogens and Antibiotic Resistance, Department of Infectious Diseases, Robert Koch Institute, Wernigerode Branch, 38855 Wernigerode, Germany; pfeifery@rki.de (Y.P.); wernerg@rki.de (G.W.); 2 Division of Infectious Diseases and Tropical Medicine, Department of Medicine II, Leipzig University Hospital, 04109 Leipzig, Germany; Sebastian.Wendt@medizin.uni-leipzig.de (S.W.); Christoph.Luebbert@medizin.uni-leipzig.de (C.L.); 3 Interdisciplinary Centre for Infectious Diseases, Leipzig University Hospital, 04109 Leipzig, Germany; Norman.Lippmann@medizin.uni-leipzig.de

**Keywords:** *Klebsiella pneumoniae*, community-acquired infection, virulence, plasmids, Germany

## Abstract

Hypervirulent *Klebsiella pneumoniae* (hvKp) is a novel pathotype that has been rarely described in Europe. This study characterizes a hvKp isolate that caused a community-acquired infection. The hypermucoviscous *Klebsiella pneumoniae* (*K. pneumoniae*) strain 18-0005 was obtained from a German patient with tonsillopharyngitis in 2017. Antibiotic susceptibility testing was performed and the genome was sequenced by Illumina and Nanopore technology. Whole genome data were analyzed by conducting core genome multilocus sequence typing (cgMLST) and single nucleotide polymorphism (SNP) analysis. Virulence genes were predicted by applying Kleborate. Phenotypic and whole genome analyses revealed a high similarity of the study isolate 18-0005 to the recently reported antibiotic-susceptible hvKp isolate SB5881 from France and the “ancestral” strain Kp52.145; both were assigned to the ST66-K2 lineage. Comparative genomic analysis of the three plasmids showed that the 18-0005 plasmid II differs from SB5881 plasmid II by an additional 3 kb integrated fragment of plasmid I. Our findings demonstrate the genetic flexibility of hvKp and the occurrence of a strain of the clonal group CG66-K2 in Germany. Hence, it emphasizes the need to improve clinical awareness and infection monitoring of hvKp.

## 1. Introduction

*Klebsiella pneumoniae* is a Gram-negative, rode shaped, facultative anaerobe, ubiquitous environmental bacterium of the family *Enterobacteriaceae*. *K. pneumoniae* is considered to be an opportunistic pathogen, mainly causing infections in immunocompromised and hospitalized patients, e.g., pneumonia, urinary tract infections, bloodstream infections and sepsis [1]. Due to the acquisition of multiple antibiotic resistance genes, like extended-spectrum beta-lactamases (ESBLs) and to a lesser extent carbapenemases, the treatment of infections with *K. pneumoniae* has become challenging [2]. The spread of various multidrug-resistant *K. pneumoniae* strains by now is considered a global public health threat [3]. In the face of increasing antibiotic resistance, a new challenge for clinicians has emerged with the rise of hypervirulent *K. pneumoniae* (hvKp) [4]. Since the mid-1980s, the emerg-ence and spread of severe hvKp infections, causing liver abscess, endophthalmitis and meningitis in otherwise healthy individuals has been reported [5]. In addition, hvKp have the ability for metastatic spread and cause infections at multiple body sites [6]. HvKp were first detected in the Asian Pacific Rim [7]. Nowadays, reports of hvKp infections are increasing worldwide. Globally disseminated hvKp strains seem to be restricted to a limited number of clonal groups (CG23, CG25, CG65, CG66 CG86 and CG380) with distinct capsular serotypes like K1 and K2 [8]. All hvKp harbor plasmids carrying genetic factors that, when expressed, enable them to escape the host immune response and support an invasive infection [4]. While the majority of serotype K1 isolates belong to CG23, serotype K2 isolates are genetically more diverse [9]. The first completely sequenced hvKp strains were NTUH-K2044 (CG23, serotype K1) and Kp52.145 (CG66, serotype K2); both have been used as reference isolates for comparative studies [10,11]. However, clinical isolates of the lineage CG66-K2 (related to Kp52.145) have only been documented twice worldwide [11]. Here we describe a community-acquired infection by a hvKp CG66-K2 isolate from a German patient.

## 2. Materials and Methods

### 2.1. Bacterial Isolate and Clinical Case

In the scope of an ongoing study on characterization of hvKp isolates diagnostic laboratories in Germany were asked to collect *K. pneumoniae* isolates with a hypermucoviscous phenotype and/or isolated from severe invasive infections. In 2018 one isolate exhibiting a hypermucoviscous phenotype was sent from a German university hospital to the Robert Koch Institute for further analysis. This isolate (no. 18-0005) was obtained in December 2017 from a throat swab of a 33-year-old male patient from Leipzig, Germany, who reported to have been engaged in unprotected oral sex practices with other men in the recent past. He presented to the outpatient infectious diseases specialist clinic because of painful tonsillopharyngitis with visible superficial ulcers on the uvula. These were treated symptomatically with octenidine mouth and throat rinse. After a few days the ulcers on the uvula were healed. Tests for HIV, hepatitis A, B and C, syphilis, chlamydia and gonococci were performed in December 2018 and March 2019; all were negative.

### 2.2. Hypermucoviscosity Phenotype Test

To verify the hypermucoviscous phenotype the isolate was subjected to the string test. A string test is defined positive when the formation of a mucoid string of >5 mm can be observed, by using a bacteriology inoculation loop to stretch a colony [12].

### 2.3. Antimicrobial Susceptibility Testing

Species identification and antibiotic susceptibility testing were performed using the broth microdilution and the automated system VITEK 2 (AST cards GN and N248); the results were interpreted according to EUCAST (European Committee on Antimicrobial Susceptibility Testing) standards and breakpoints vers. V10 (http://www.eucast.org/clinical_breakpoints).

### 2.4. Whole Genome Sequencing (WGS) and De Novo Assembly

DNA was extracted from overnight cultures in Brain Heart Infusion (BHI) broth using DNeasy Blood & Tissue Kit (Qiagen) and MagAttract Kit (Qiagen, Hilden, Germany) in line with the manufacturer’s instructions. The DNA was quantified by applying the Qubit dsDNA HS Assay Kit (Invitrogen/Thermo Fisher Scientific, Karlsruhe, Germany). Sequencing libraries for short read sequencing were prepared applying the Nextera XT Kit (Illumina, San Diego, CA, USA) and sequenced on an Illumina Miseq using v3 chemistry (2 × 300 bp) according to the manufacturer’s protocol. Read qualities were assessed using the pipeline QCumber (v 2.1.1) where the FastQC (v 0.11.5) and Kraken tools are implemented. Raw reads were trimmed by Trimmomatic (v 0.36; options ‘sliding window 4:20′) and de novo assembled by the SPAdes (v 3.12.0) algorithm with default parameters. The isolate was also subjected to long-read sequencing by MinION (Oxford Nanopore Technologies, Oxford, UK). A hybrid assembly of Illumina and MinION sequence data was done by using Uniclyer (v.0.4.4).

Genome sequences were submitted to NCBI SRA and are available under BioProject accession no PRJNA678637.

### 2.5. WGS-Based Typing and Virulence and Resistance Gene Prediction

Multilocus sequence typing (MLST) and cgMLST were performed by applying the Ridom software SeqSphere+ (v7.1.0, Ridom GmbH, Münster, Germany). Single nucleotide polymorphisms (SNPs) were analyzed by using SNPfilter (https://gitlab.com/s.fuchs/snpfilter). Based on the aligned variant positions a maximum-likelihood tree was calculated by using PhyML (v3.1). The maximum-likelihood tree was visualized by using the iTOL software (v5.6.1). Identification of capsule types (K types) was done by using Kaptive (http://kaptive.holtlab.net/). The presence of known virulence factors (*clb*, *ybt*, *iro*, *iuc*, *rmpA/rmpA2*) and various resistance genes was determined by applying the Kleborate tool (https://github.com/katholt/Kleborate) [8,13,14].

## 3. Results and Discussion

**Figure 1:**
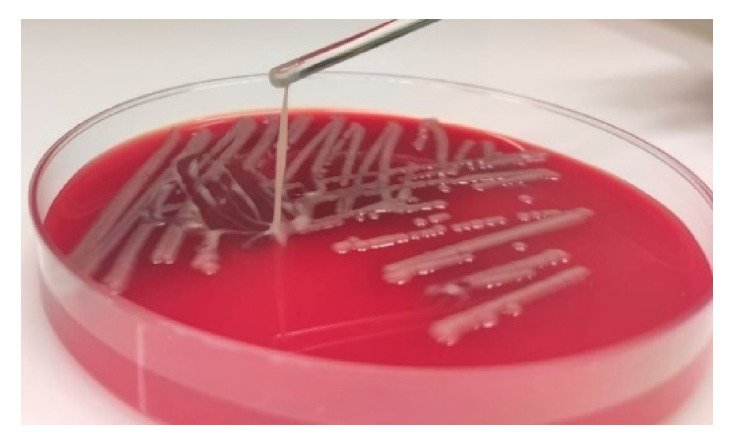
Hypermucoviscous phenotype of the hvKp strain 18-0005. The hypermucoviscous phenotype is represented by a positive “string test”.

In the reported case, a 33-year-old male patient presented a tonsillopharyngitis caused by a community-acquired hvKp strain. Although pyogenic liver abscess is the hallmark clinical syndrome, the clinical picture of hvKp infections is diverse and pharyngitis has been described before occasionally [7,15]. After treatment with octenidine mouth and throat rinse the patient remained clinically asymptomatic without disseminated infections. This suggests that an early detection and treatment of hvKp infections may be important to prevent an invasive progression. Diabetes, alcoholism and HIV are host risk factors that have been associated with hvKp infections [4]. However, the medical history of the patient showed no underling diseases. The analyzed isolate 18-0005 presented a hypermucoviscous phenotype, that is one of the defining features of hvKp strains (Figure 1) [16]. Although the direct association of hypervirulence with hypermucoviscosity is controversial, this case demonstrates that a positive string test is a strong indicator for presence of a hvKp strain. Isolate 18-0005 was fully susceptible to all tested antibiotics, which agrees with previous studies on hvKp (Table 1) [17].

**Figure 2:**
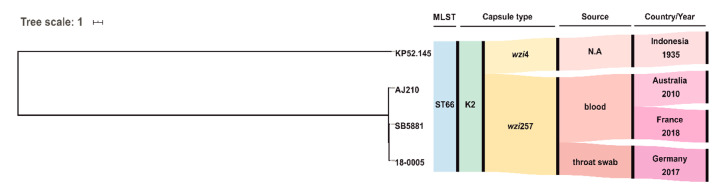
Phylogeny of the four hvKp strains Kp.52.145, AJ210, SB5881 and 18-0005 (this study). The maximum likelihood tree was calculated by PhyML based on single nucleotide polymorphisms (SNPs) filtered after mapping to the Kp52.145 sequence. N.A. not available

MLST analysis of the SPAdes assembled genome of 18-0005 identified sequence type ST66 that belongs to the clonal group CG66 [6]. Capsule typing revealed the serotype K2. Rodrigues et al. recently described the first ST66-K2 isolate identified in Europe; this isolate was obtained from blood and urine samples of a patient infected with HIV in 2018 in France [11]. Phylogenetic analysis revealed a relationship to the three known ST66-K2 isolates: (I) SB5881 isolated in 2018 from France [11], (II) AJ210 isolated in 2010 from Australia [18], and (III) “ancestral” reference Kp52.145 detected in 1935 in Indonesia (Figure 2) [11,19]. However, 18-0005 was more closely related to SB5881 (84 SNPs) than to AJ210 (152 SNPs) or Kp52.145 (409 SNPs).

**Figure 3:**
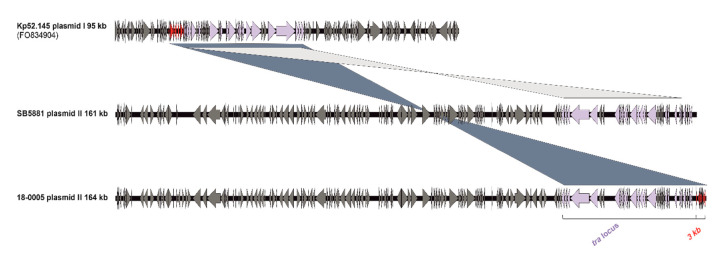
Comparison of plasmid II sequences of hvKp strain 18-0005 with previously described plasmid II sequences of strain SB5881 and reference strain Kp52.145 (plasmid I). Based on a pairwise BLAST analysis (blastn) of the sequences and visualized by the program Easyfig (v.2.2.2). ORFs are illustrated as arrows. The shared *tra* locus (light purple) and the new integrated 3 kb fragment (red) are coloured.

Kleborate analysis identified the virulence determinants aerobactin (*iuc*_2), salmochelin (*iro*_2) and the regulator of mucoid phenotype (*rmpA_*9/*rmpA2*_9). While Kp52.145 and AJ210 harbored only plasmid I (95 kb) and plasmid II (121 kb) a third small plasmid (3.7 kb) was detected in SB5881 and 18-0005. Furthermore, the latter two strains each carried a larger plasmid II. Comparison of the plasmid sequences in all isolates revealed the same integrated fragment on plasmid I, encoding a conjugation machinery; a copy of this fragment was detected on plasmid II in isolates SB5881 and 18-0005 (Figure 3). However, different from SB5881 plasmid II (161 kb), isolate 18-0005 harbored an additional 3 kb fragment of plasmid I in plasmid II (164 kb). This fragment contained putative transcription regulators and several uncharacterized proteins. These findings suggest a genetic flexibility of the virulence plasmid to integrate further virulence or antibiotic resistance determinants, in addition to its possibility of a conjugative transfer.

## 4. Conclusions

While hvKp has undergone an epidemic spread in Asian countries, reports of community-acquired infections in European countries remained scarce. Hence, hvKp are underrecognized due to the lack of awareness of hvKP among clinical laboratories and missing reliable diagnostic tests. The reported case demonstrates the occurrence of a hvKp strain of the worldwide rarely described CG66-K2 lineage in Germany. Although the plasmid II is stably maintained in this lineage, our findings reflect that genetic rearrangements can occur by the insertion of a large plasmid I fragment (40–43kb). The attendance of plasmid II to integrate DNA possesses a potential risk for the emergence of convergent virulent and antibiotic-resistant ST66-K2 variants.

## Figures and Tables

**Table 1:** Antibiotic susceptibilities of hypervirulent *K. pneumoniae* strain 18-0005.

Antibiotic	MIC (mg/L)	Interpretation
Piperacillin	≤4	S
Piperacillin/Tazobactam	≤4	S
Cefotaxime	≤1	S
Ceftazidime	≤1	S
Cefepime	≤1	S
Aztreonam	≤1	S
Imipenem	≤0.25	S
Meropenem	≤0.25	S
Amikacin	≤2	S
Gentamicin	≤1	S
Tobramycin	≤1	S
Ciprofloxacin	≤0.25	S
Moxifloxacin	≤0.25	S
Tigecycline	≤0.5	S
Fosfomycin	≤16	S
Colistin	≤0.5	S
Trimethoprim/Sulfamethoxazole	≤2	S

MIC minimum inhibitory concentration; S susceptible.

## Data Availability

The Genome sequences were submitted to NCBI SRA and are available under BioProject accession no PRJNA678637.
